# Menthol Inhibits *Candida albicans* Growth by Affecting the Membrane Integrity Followed by Apoptosis

**DOI:** 10.1155/2022/1297888

**Published:** 2022-10-28

**Authors:** Gajanan Zore, Archana Thakre, Mazen Abdulghani, Kajal Bhosle, Amruta Shelar, Rajendra Patil, Kiran Kharat, Sankunny Karuppayil

**Affiliations:** ^1^School of Life Sciences, Swami Ramanand Teerth Marathwada University, Nanded 431606, MS, India; ^2^Taiz University, Taiz, Yemen; ^3^Department of Biotechnology, Savitribai Phule Pune University, Pune 7, MS, India; ^4^Department of Biotechnology, Deogiri College, Aurangabad, MS, India; ^5^Center for Interdisciplinary Studies, Department of Medical Biotechnology, Stem Cell and Regenerative Medicine, DYPU, Kolhapur, Maharashtra, India

## Abstract

Inclusion of *Candida albicans* in the list of pathogens with potential drug resistance threat in recent years has compelled scientists to explore novel and potent antifungal agents. In this study, we have evaluated anti-*Candida* potential of menthol against different growth forms and synergistic potential with fluconazole. Menthol inhibited planktonic growth of all the isolates completely at ≤3.58 mM and killed 99.9% inoculum at MIC, indicating that menthol is fungicidal. Menthol inhibited hyphal form growth completely at 0.62 mM. It has inhibited developing a biofilm by 79% at 3.58 mM, exhibiting excellent activity against recalcitrant biofilms. FIC index values of 0.182 and 0.093 indicate excellent synergistic activity between fluconazole and menthol against planktonic and biofilm growth, respectively. Menthol enhanced rate of OxPhos among 22% cells; arrested 71% cells at G2-M phase of cell cycle and induced apoptosis in 15% cells. Thus, menthol exhibits excellent anti-*Candida* activity against differentially susceptible isolates as well as various growth and morphological forms of *C. albicans*. Menthol affects membrane integrity thereby inducing oxidative stress followed by cell cycle arrest and apoptosis. Considering the excellent anti-*Candida* potential and as it is Generally Recognized as Safe by the Food and Drug Administration, it may find use in antifungal chemotherapy, alone or in combination.

## 1. Introduction

The *Candida* species is the fourth most common pathogen responsible for life-threatening invasive candidiasis including candidemia and nosocomial infections in the form of biofilms exhibits very high mortality (40–50%) [1, 2]. It is considered as difficult-to-treat infections especially among immunocompromised individuals [2, 3]. Morphophysiological plasticity enables *C. albicans* cells to respond differentially towards host defense mechanisms and chemotherapeutic agents [2, 3]. *Candida* species is included in the list of pathogen with the potential drug resistant threat by CDC, recently in consultation with other institutions, considering its current and projected impact in clinics and on economy [4]. Thus, considering the promise plant-derived agents has shown against various infectious and noninfectious diseases, plant-derived anti-*Candida* agents are being explored on highest priority worldwide [5].

Menthol, a cyclic monoterpene alcohol, is one of the major active ingredients of peppermint (*Mentha canadensis* L) [6]. Peppermint plants are being used in traditional systems of medicine worldwide from centuries, long before identification of menthol in eighteenth century and were reported to exhibit various biological activities like antimicrobial, anticancer, immunomodulatory, anti-inflammatory, antiviral, etc. [7, 8]. Menthol was isolated and identified by Gambius (1771). It exists in L (+) or (−) forms naturally. It is one of the most frequently used flavoring agent in pharmaceuticals, cosmetics, pesticides, liqueurs, toothpastes, shampoo, tobacco, etc. [6, 8]. Menthol exhibits various biological activities like it binds and activate TRPM8 (transient receptor potential cation channel) that leads to increase in calcium concentration and induce cold response, causes the vasodilation of cutaneous microvasculature and thus is being used as a very good enhancer of drug uptake in various topically applied analgesics [9]. Insecticidal activity of peppermint oil against *Culex quinquefasciatus*, *Aedes aegypti*, and *Anopheles tessellatus* was ascribed to menthol [10]. Derivatization of menthol into menthyl cinnamate and menthyl chloroacetate significantly enhanced the mosquitocidal activity of menthol [10].

Antimicrobial (antibacterial and antifungal) activity of menthol as well as synergistic activity with antibiotics, like oxacillin, norfloxacin, or erythromycin, against bacterial and fungal pathogens were reported in several recent studies [8]. However, the mechanism of antimicrobial activity is not understood. Trombetta et al. suggested that membrane solubilizing activity of monoterpene could be associated with antibacterial activities. Menthol exhibited excellent antifungal activity against various plant and human pathogenic fungi at very low concentrations [11, 12]. Anti-*candida* activity of menthol is reported recently and antifungal activity was found to be isomer-dependent, i.e., (+)-menthol and (−)- menthol were most effective ones exhibiting MIC 1.50 mM [13]. However, the mechanism of antifungal activity is not known. The present study is aimed at evaluating anti-*Candida* activity of menthol against different growth forms viz. planktonic, biofilm, and hyphae. We have also made an attempt to understand mechanism of anti-*Candida* activity by cell cycle, MMP, real-time qPCR, and apoptosis assay. Our results provide an insight into the mechanism of anti-*Candida* activity of menthol.

## 2. Materials and Methods

### 2.1. Plant Molecules and Growth Media

Menthol (97%) was purchased from Sigma-Aldrich India Pvt. Ltd., Bangalore (India). Yeast extract peptone dextrose broth, RPMI 1640 medium, horse Serum, and MTT were purchased from Hi-Media Laboratories, Pvt. Ltd., Mumbai (India). Polystyrene 96 well microtiter plates were procured from Tarson India Ltd.

### 2.2. *Candida albicans* Isolates and Strains

Fourteen clinical isolates and two strains of *Candida albicans*, differentially susceptible to fluconazole (FLZ) (Resistant-*N* = 6, S-DD-*N* = 02 and susceptible-*N* = 8) used in this study were isolated from sputum, blood, cerebrospinal fluid (CSF), ascitic fluid, pus and stool [14]. Clinical isolates were received from Swami Ramanand Teerth Culture Collection (SRTCC) of School of Life Sciences, Swami Ramanand Teerth Marathwada University, Nanded (MS) India. A fluconazole-resistant strain of *C. albicans* ATCC 10231 [15, 16], and a susceptible strain ATCC 90028 were procured from the Microbial Type Culture Collection, Institute of Microbial Technology (IMTECH), Chandigarh (India), and included as a quality control in this study. All the cultures were maintained on yeast extract peptone dextrose (YPD) agar slants at 4°C [16].

### 2.3. Inhibition of Planktonic Growth of *Candida albicans*

#### 2.3.1. Inoculum Preparation

*C. albicans* cells grown in YPD for 24 h at 30°C were harvested, washed with sterile distilled water, and resuspended in phosphate buffered saline (PBS). Cell density was adjusted to 2 × 10^3^ cells/ml, aseptically and used in following experiments.

#### 2.3.2. Broth Microdilution Assay

Anti-*Candida* activity of menthol was tested with different concentration (from 0.22 mM to 28.6 mM) against fourteen clinical isolates and two standard strains of *C. albicans* differentially susceptible to fluconazole (Resistant-*N* = 6, S-DD-*N* = 02 and susceptible-*N* = 8) by using a broth microdilution assay as mentioned previously with slight modification as per Wayne and Thakre et al. [16–18]. The lowest concentration required for complete growth inhibition (no visible growth) was determined as minimum inhibitory concentration (MIC) [18]. Triplicates were used for each concentration and the experiment was repeated thrice. 5 *µ*l cultures from these wells were inoculated on YPD agar plates and number of colonies appeared on the agar plates after 48 h of incubation at 35°C were counted. Lowest concentration required for killing of 99.9% inoculums was considered as MFCs [16, 17].

#### 2.3.3. Time Kill Assay

Time-dependent killing of *C. albicans* (ATCC 10231) inoculums by menthol at MFC was studied as described previously by Zore et al. and Thakre et al. [14, 16, 19]. In brief, 500 *µ*l of *C. albicans* inoculum (prepared in YPD broth medium) containing 3.58 mM (MFC) of menthol was aliquoted in 1.5 ml vials. Vials were incubated at 35°C. Cells were harvested at different time intervals (0, 5, 10, 20, 40, 80, and 160 min), washed to remove any drug carryover and resuspended in 100 *µ*l of PBS. 50 *µ*l of these cells were inoculated on YPD agar plates and number of colonies appeared were counted after 48 h of incubation and compared with control plates. Triplicate vials were harvested after each time interval, vials without menthol were used as controls, and the experiment was repeated thrice.

### 2.4. Inhibition of Morphogenesis in *Candida albicans* (ATCC 10231)

Effect of menthol (28.6 to 0.44 mM) on serum-induced morphogenesis of *C. albicans* (ATCC 10231) was studied by using a microtiter plate-based morphological assay as done previously by Zore et al. and Thakre et al. [14, 16, 19]. In brief, 100 *µ*l of inoculum (2 × 10^5^ cells/ml) prepared in YPD broth-containing horse serum (20%) was distributed in each wells of a 96 well microtiter plate. Menthol (28.6 to 0.44 mM) was added to each well as a doubling dilutions and incubated at 37°C for 90 min. Cells of different morphological types (budded, unbudded, hyphae, and pseudohyphae) were counted after incubation microscopically using hemocytometer. Percentage inhibition of hyphae induction was calculated by comparing with control. Wells without menthol were used as controls. Each concentration was tested in triplicates and the experiment was repeated thrice.

### 2.5. Inhibition of Biofilm (Development and Maturation) Formation

#### 2.5.1. Inoculum Preparation

*C. albicans* cells grown in YPD for 24 h at 30°C were harvested, washed with sterile distilled water, and resuspended in phosphate buffered saline (PBS). Cell density was adjusted to 1 × 10^7^/ml, aseptically [20].

#### 2.5.2. Biofilm Assay

Effect of menthol (1.79–57.3 mM) was evaluated by measuring, development (24 h), and maturation (48 h) of *C. albicans* (ATCC 10231) biofilms by MTT assay after removing non-adhered cells as done previously by Thakre et al. [16, 20] and suggested by Ramage et al. [21]. In brief, adhesion was performed by adding 100 *µ*l of inoculum aseptically in the wells of 96 well plate and incubated at 37°C for 90 min. After incubation, each well was washed thrice using sterile PBS to remove non-adhered cells. 100 *µ*l of fresh RPMI 1640 medium was added in each well of a prewashed microtiter plate and incubated at 37°C for 24 h for biofilm development. A developing biofilm was observed after washing and removing non-adhered cells. For a mature biofilm, developing biofilms were further incubated till 48 h by adding fresh medium after washing and removing non-adhered cells. Respective doubling dilutions (1.79–57.3 mM) of menthol were added to the wells of 96 well microtiter plates while adding the fresh medium, i.e., for a developing biofilm after 90 min of incubation and for a mature biofilm after 24 h incubation. Control wells were lacking menthol. Triplicates were used for each concentration and experiment was repeated three times. MTT assay was performed as described previously by Thakre et al. [16, 20].

#### 2.5.3. Synergistic Assay

The effect of menthol on fluconazole susceptibility in planktonically growing *C. albicans* (ATCC 10231) cells was studied by a microdilution checkerboard assay as done previously Thakre et al. and Odds [16, 17, 22]. Concentrations of menthol and fluconazole used were 0.22 to 7.16 mM and 2 to 128 mg/L, respectively. Similarly, the synergistic activity of fluconazole (≥256-8 *µ*g/ml) and menthol (0.22 to 7.16 mM) was evaluated against development and maturation of *C. albicans* biofilms as done previously. Fractional inhibitory concentrations (FICs) and FIC index values were calculated for menthol as per Odds [22]. FIC is inhibitory concentration in combinations divided by the concentration that has same effect when used individually while FIC index values are sum of the FICs. The FIC index (∑ FIC) value shows the type of interaction between two compounds viz. value 0.5 to 4.0 shows additive interaction while a value less than 0.5 indicates synergistic interaction [22].

#### 2.5.4. Mitochondrial Membrane Potential Assay

Effect of menthol on mitochondrial membrane potential was evaluated using MitoTracker® Red dye and Alexa Fluor® 488 annexin V (Component A) in a flow cytometer as done previously by Thakre et al. [20]. In brief, *C. albicans* cells (5 × 10^6^ cells/ml) grown for 24 h at 30°C were exposed to menthol (MIC50) for 6 h. After incubation, 1 *μ*l of the MitoTracker® Red dye (10 mM) was added to each well and incubated for 30 minutes at 37°C in an atmosphere of 5% CO_2_. Cells were washed with PBS and resuspended into 100 *μ*l of 1X annexin-binding buffer. 5 *μ*l of Alexa Fluor® 488 annexin V (Component A) was added into each well and incubated for 15 min at room temperature. Finally, 400 *μ*l 1X annexin-binding buffer was added in each well and stained cells were analyzed using a flow cytometer by measuring the fluorescence emission at 530 nm and 585 nm.

#### 2.5.5. Cell Cycle Assay

Starved (1 h) cells of *C. albicans* (2 × 10^3^ cells/ml) were exposed to subtoxic concentration of menthol (1 mM) for 24 h at 30°C and used for cell cycle analysis using Attune flow cytometry (Invitrogen, USA) as described previously by Thakre et al. and Zore et al. [17, 19]. In brief, inoculum of *C. albicans* treated with menthol (1 mM) for 24 h in YPD broth. After incubation, cells were harvested and washed twice using 1 ml of 50 mM Tris pH 7.8. These cells were treated with 10 *µ*g of RNase A in 500 *µ*l of 50 mM Tris and incubated for 2 h at 37°C, followed by addition of protease (5 mg/ml pepsin in 0.05 M HCl) for 1 h at 25°C. Thereafter, cells were harvested and resuspended in 500 *µ*l FACS assay buffer (200 mM Tris/HCl pH 7.5; 200 mM NaCl; 78 mM MgCl_2_). Finally, 15 *µ*l of propidium iodide (1 mg/ml) was added and incubated for 30 min, and the cell cycle was monitored using flow cytometry.

#### 2.5.6. Apoptosis Assay

Menthol (MIC50)-induced apoptosis in *C. albicans* cells was assessed after 24 h of incubation using Annexin V/Alexa Fluor®568 conjugate/PI binding and flow cytometry analysis as described previously by Thakre et al. [17]. Samples were used in triplicates and the experiment was repeated thrice.

### 2.6. Real-Time qPCR Analysis of Selected Genes

Total RNA was extracted using RNeasy Mini kit (50 reaction) (Cat. No. 74104, Qiagen Pvt. Ltd.) as per the manufacturers' instructions using lyticase for cell lysis from the *C. albicans* cells (1 × 10^7^) exposed to menthol. Then, cDNA synthesis was carried out using 2 *µ*g total RNA and High-Capacity cDNA Reverse Transcription Kit (Applied Biosystems Cat. No. 4368814) as per the manufacturers' instructions. The expression analysis of three genes was carried out using KAPA SYBR® FAST qPCR Kit Master Mix (2X) Universal and using a CFX96 Touch™ Real-Time PCR Detection System (Bio-Rad Pvt. Ltd) as per manufacturers' instructions and parameters. Triplicates were run using biological replicates and data are reported as mean ± S.D. Statistically significant was calculated using ANOVA and *p* values ˂0.05 were considered statistically significant. The gene expression was normalized first to GAPDH levels and then to untreated control cells. The primers used in this study are shown in Table 1.

## 3. Results

### 3.1. Inhibition of Planktonic and Hyphal Form Growth

Antifungal potential of menthol against different morphological (planktonic and hyphal) and growth (biofilm) forms of *C. albican*s isolates and strains (*n* = 16) is evaluated in present study. Among the morphological forms, the hyphal form was found more sensitive compared to planktonic growth. Menthol inhibited planktonic growth of all the clinical isolates and strains differentially susceptible to fluconazole (Resistant-*N* = 6, S-DD-*N* = 02 and susceptible-*N* = 8) tested in this study at or below 3.58 mM (Table 2). Among the sixteen isolates and strains tested, eleven required 3.58 mM menthol for complete growth inhibition, while five were inhibited completely only at 1.79 mM (Table 2).

**Figure 1:**
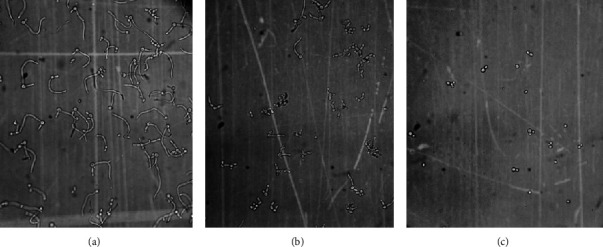
Light microscopic (40x) images of *C. albicans* cells. (a) Cells growing in the absence of menthol (control), (b) cells growing in presence of methanol (0.62 mM), and (c) cells growing in presence of methanol (1.79 mM).

**Figure 2:**
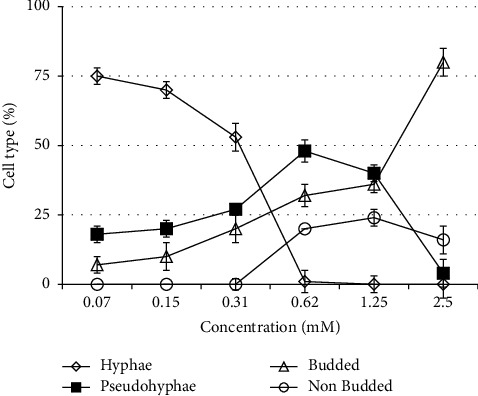
Effect of menthol on morphogenesis of *C. albicans*.

**Figure 3:**
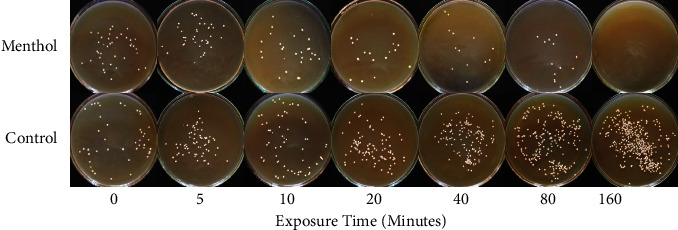
Time-dependent killing of *C. albicans* cells in response to MFC (3.58 mM) of menthol.

Hyphal form growth was more sensitive to menthol than that of planktonic growth. Menthol inhibited serum (20%)-induced hyphae induction completely at 0.62 mM concentration (Figures1(a)–1(c) and 2). To confirm whether menthol is fungistatic or fungicidal, MFC (minimum fungicidal concentration) assay was carried out. It showed that menthol was fungicidal at MIC for all the isolates and strains tested in this study (Table 2). To determine the time required for exerting fungicidal effect, a kill curve analysis of menthol at MFC was performed. Results of the kill curve analysis showed time-dependent killing of inoculums as MFC of menthol killed 99.9% inoculums within 160 min of exposure (Figure 3).

### 3.2. Inhibition of Biofilm Growth

**Figure 4:**
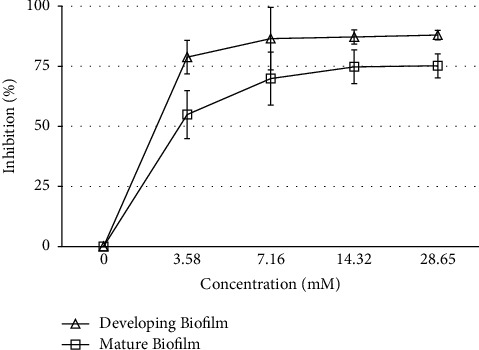
Inhibition of *C. albicans* biofilm progression by menthol.

Considering the excellent activity of menthol against planktonic and hyphal form growth, we have evaluated effect of menthol onstage biofilm growth at different stages, i.e., a developing and mature biofilm. The MIC of planktonic growth (3.58 mM) inhibited developing and mature biofilm by 79% and 55%, respectively (Figure 4, Table 2). The developing biofilm was found to be more sensitive as 7.16 mM menthol caused 84% inhibition compared to 70% in case of a mature biofilm (Figure 4, Table 2). Doubling the concentration to 14.32 mM could achieve inhibition of 87% in case of developing and 75% in case of mature of biofilm growth and further increase in concentration could not increase the % inhibition, significantly (Figure 4, Table 2). Our result suggests that menthol exhibit excellent activity against *C. albicans* biofilm.

### 3.3. Synergistic Activity of Menthol with Fluconazole against *C. albicans* ATCC 10231

Synergistic activity of menthol with fluconazole against planktonic and biofilm growth of a fluconazole resistant strain of *C. albicans* (ATCC 10231) was evaluated using checkerboard assay. Menthol (0.44 mM) sensitized and brought down MIC of fluconazole from 128 mg/L to 8 mg/L for planktonic growth (Table 2). The FIC index value of 0.182 indicates excellent synergistic activity of menthol with fluconazole against planktonic growth (Table 2). Similarly, menthol (1.79 mM) brought down MIC 90 of a developing biofilm and MIC 70 of a mature biofilm to 8 mg/L from 256 mg/L fluconazole (Table 2). The FIC index value of 0.093 indicates excellent synergy between fluconazole and menthol against recalcitrant biofilm growth of a fluconazole-resistant strain of *C. albicans* ATCC 10231 (Table 2).

#### 3.3.1. Mitochondrial Membrane Potential Assay

**Figure 5:**
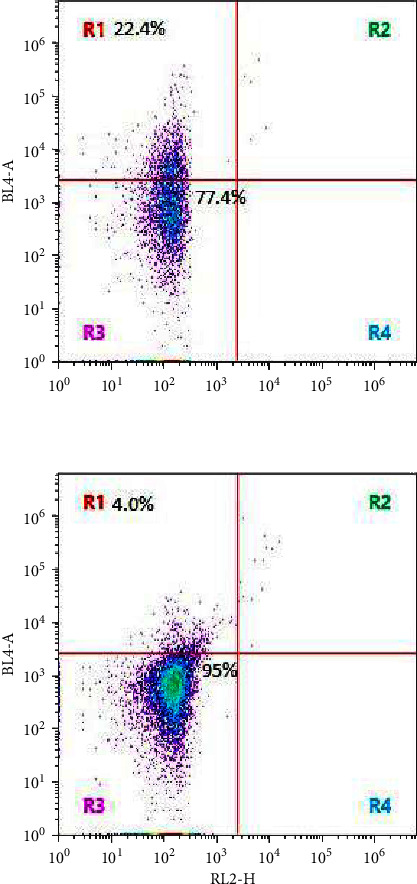
Mitochondrial membrane potential of *C. albicans* in presence of (upper) menthol (1 mM), (lower) control. Quadrant analysis of fluorescence intensity of gated cells in RL2-H (Alexa Fluor® 488 annexin V (component A) and BL4-A (PI) channels was from 10,000 events. Values shown were percentages of each quadrant. ^*∗*^*p* < 0.05, in comparison to control.

Menthol is known to modulate membrane permeability and induce membrane damage stress. We have evaluated the effect of menthol on *C. albicans* mitochondrial membrane using a mitochondrial membrane potential assay. Our results showed that menthol (1 mM) at subtoxic concentration enhanced oxidative phosphorylation by 22.4% compared to 4% in control (Figure 5).

#### 3.3.2. Cell Cycle and Apoptosis Assay

**Figure 6:**
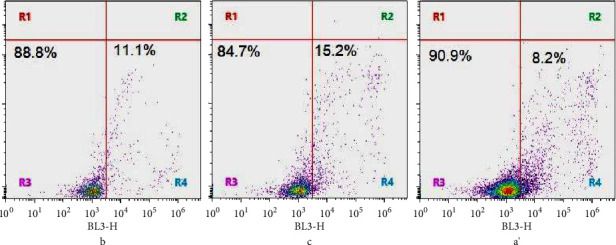
Induction of apoptosis in *C. albicans* ATCC10231 cells, where (a) Untreated *C. albicans* cells; (b) *C. albicans* cells treated with menthol (0.5 mM); and (c) *C. albicans* cells treated with menthol (1 mM). Menthol-treated apoptotic Candida albicans cells were stained with annexin V/PI and subjected to flow cytometry analysis. The four quadrants represent living cells R3 (annexin V-PI-), early apoptotic R4 (Annexin V + PI-), late apoptosis R2 (annexin + PI+) or necrotic or dead R1 (annexin V-PI+) stages. Quadrant analysis of fluorescence intensity of gated cells in BL3-H (annexin V-FITC) and BL1H (PI) channels was from 10,000 events. Values shown were percentages of each quadrant. ^*∗*^*p* < 0.05, in comparison to control.

Membrane damage stress is reported to exert oxidative stress followed by cell cycle arrest and apoptosis. In this study, our cell cycle analysis suggests that at subtoxic concentration (1 mM), menthol arrests 71% cells in G2-M phase, compared to 57% in control (Table 3). It indicates an apoptosis-inducing potential. We have confirmed apoptosis inducing potential of menthol using apoptosis assay based on Annexin V Alexa Fluor® 568 conjugate/PI binding and flow cytometry. Flow cytometry analysis showed that subtoxic concentrations, i.e., 0.5 and 1 mM of menthol induce apoptosis in 11.1% and 15.2% cells of *C. albicans,* respectively, compared to 8.2% in control (Figures 6(a)–6(c)).

### 3.4. Real-Time qPCR Analysis of Selected Genes

**Figure 7:**
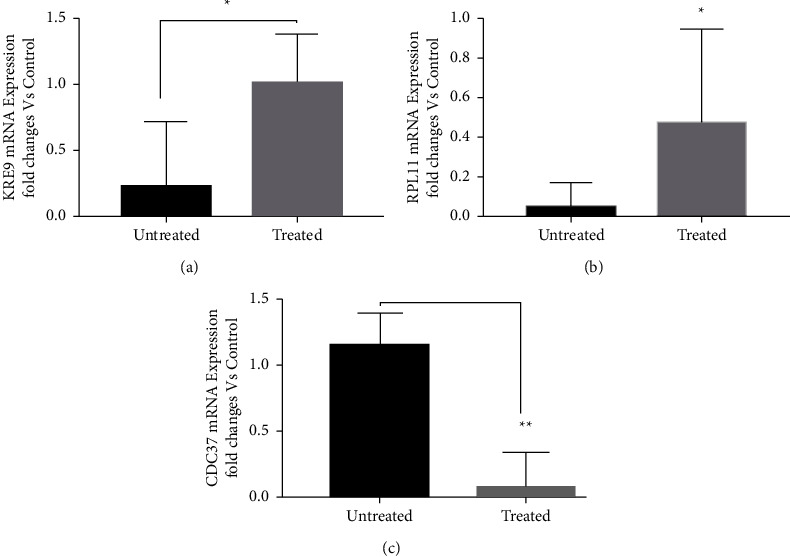
Expression analysis of selected genes of *Candida albicans* using real-time qPCR (*KRE9, RPL11* and *CDC37*) in response to menthol. mRNA copy was normalized initially to GAPDH levels and later to the untreated control mRNA copy number. Data are expressed as mRNA copies in cells, where significance referrers to the difference between untreated control and treated cells (*n* = 3, ^*∗∗*^*p* < 0.01, ^*∗*^*p* < 0.05). Bar indicates mean, and error bar indicates SD.

The expression analysis of three genes viz., *KRE9, RPL11*, and *CDC37* was carried out using real-time qPCR to support our study. Out of the three, two were upregulated while one was downregulated as compared to control. The expression pattern of three genes is shown in (Figure 7).

## 4. Discussion

Menthol is a cyclic monoterpene alcohol found in peppermint (*Mentha canadensis* L) and exists in L (+) or (−) forms naturally. It is a major active principle and thus is reported to be associated with most of the biological activities of peppermint like antimicrobial, anticancer, immunomodulatory, anti-inflammatory, antiviral, etc., to cite a few [6–8]. Monoterpene molecules, in addition to multifarious biological activities, were reported to enhance membrane permeability and transdermal uptake of several drugs [23]. Menthol is considered as a better and safer enhancer of transdermal drug uptake than other monoterpene molecules as it does not induce convulsions in humans in response to overdose and thus being used as an adjuvant [23, 24]. Though membrane destabilizing activity of monoterpenes is hypothesized to cause depolarization and inhibition of microbial growth, the mechanism of anti-*Candida* activity of menthol is not understood fully.

Menthol demonstrated differential activity against different morphological and growth forms of *C. albicans* in our study. Menthol inhibited planktonic growth and hyphae induction completely at 3.58 and 0.62 mM concentrations, respectively, while 7.16 mM concentration could inhibit growth of a developing biofilm by 84% and mature biofilm by 70%, only (Table 2, Figure 4). The differential activity could be associated with membrane destabilizing activity of menthol. Membrane, in addition to encapsulating the cellular components, also plays a very important role in cellular signaling. Signaling pathways (MAPK, cAMP-PKA, and HOG1) regulate morphophysiology of *C. albicans* cells in response to environmental cues [3, 25]. Membrane properties are highly dynamic and reported to depend on morphological and growth forms of *C. albicans* [26]. Terpene-induced membrane integrity damage is reported to induce oxidative stress, enhance fluconazole uptake, and affect signaling pathways regulating hyphae induction, previously [14, 16, 17, 19]. Thus, membrane destabilizing activity of menthol could be affecting signaling pathways, thereby inhibiting hyphae induction in *C. albicans* at very low concentration.

The difference in efficacy of menthol could be due to the difference in bioavailability of menthol under planktonic and biofilm form growth [27]. Biofilms are made up of adherent microbial cells colonizing living surfaces or tissues embedded in an extracellular matrix [28]. It is a highly structured growth form composed of yeast, pseudohyphae, and hyphal cells and thus exhibits distinct properties, making biofilms recalcitrant to antifungal agents as well as immune responses [29]. Menthol inhibited biofilm growth significantly, i.e., more than 70%. Extracellular matrix, metabolic plasticity, and upregulation of drug efflux pumps are believed to confer resilience toward antifungal agents and immune responses [30, 31]. The *β*-glucan present in extracellular matrix is reported to protect *C. albicans* cells from neutrophil attacks [32]. FIC index values suggest that menthol exhibits excellent synergistic activity with fluconazole against planktonic (0.182) and biofilm growth (0.093). Membrane-destabilizing activity of menthol could enhance bioavailability of fluconazole in a fluconazole-resistant strain used in our study and reduce MIC of fluconazole against both planktonic and biofilm growth. As menthol is reported to enhance membrane permeability, we conclude that synergistic activity of menthol with fluconazole is a result of membrane destabilizing activity of menthol. Menthol is known for increasing membrane permeability and drug uptake; thus, it is being used as an adjuvants [8]. Thus, menthol may find use in antifungal chemotherapy alone or as an adjuvant and reduce side effects associated with higher or prolonged use of chemotherapeutic agents especially against drug-resistant isolates and difficult-to-treat infections like biofilms [33]. Membrane-destabilizing activity of monoterpenes is also reported to induce leakage of intracellular components and inhibit microbial growth [11].

In addition to permeability, terpene-induced cell wall and membrane integrity damage is reported to exert oxidative stress in microorganisms including *C. albicans* [11, 14, 17, 19]. Our real time qPCR analysis revealed that *KRE9* was significantly upregulated in response to menthol compared to control (Figure 7). *KRE9* gene encodes an O-glycoprotein involved in assembly of 1–6, beta glucan in the cell wall and was reported to be upregulated in response to limonene-induced cell wall and membrane damage [17]. Upregulation of *KRE9* suggests menthol-induced cell wall and membrane damage. It was further supported by our MMP assay wherein, menthol modulated membrane potential (Figure 5). Modulation of membrane potential by menthol is well documented; wherein, it was reported to accelerate inactivation of L-type (high-threshold) Ca^2+^ currents (activated from a holding potential of −80 mV to positive potentials above −20 mV). Menthol inhibits Ca^2+^ influx through the low voltage-activated Ca^2+^ channel, and enhances the inactivation of the L-type (high voltage-activated) Ca^2+^ channel [34, 35]. Our MMP assay confirms increased membrane potential in *C. albicans*.

As terpene-induced membrane and cell wall damage is reported to generate ROS and thus oxidative stress that often induce DNA damage in *C. albicans* [17, 19]. Upregulation of *RPL11* in response to menthol indicates nucleolar stress. *RPL11* is an indicator of nucleolar stress caused by DNA damage induced by oxidative stress. Our earlier studies have shown that oxidative stress caused by membrane and cell wall damage in *C. albicans* by limonene and piperine exerts nucleolar stress followed by cell cycle arrest and apoptosis [17, 20]. Menthol downregulated *CDC37* an essential chaperone reported to be involved in cell cycle regulation in our RT-qPCR analysis. *CDC37* is reported to be indispensable for cell growth as heterozygous mutants were susceptible to chemicals while homozygous null mutants were inviable. Thus, downregulation of *CDC37* by menthol confirms cell cycle arrest in *C. albicans*. It was further confirmed using cell cycle and apoptosis assay; wherein, menthol arrested *C. albicans* cells at G2-M phase and induced apoptosis.

In general, our data suggest that membrane-destabilizing activity of menthol is associated with differential inhibition of *C. albicans* growth (planktonic, biofilm, and hyphae) as membrane properties varies according to morphological forms. Modulation of mitochondrial membrane potential, overexpression of *KRE9 (*cell wall and membrane damage*), RPL11* (nucleolar stress), and downregulation of *CDC37* (cell cycle) genes confirm membrane damage stress inducing ROS followed by nucleolar stress, cell cycle arrest, and apoptosis. Demonstration of cell cycle arrest and apoptosis by flow cytometry-based assays further confirms that menthol inhibit *C. albicans* growth by destabilizing membrane causing oxidative stress that leads to nucleolar stress followed cell cycle arrest and apoptosis.”

## 5. Conclusion

Our data suggest that menthol exhibits considerable anti-*Candida* activity against various growth forms alone or in combination with fluconazole. Based on our findings, we conclude that menthol-induced membrane and cell wall destabilization exert oxidative stress (ROS), leading to DNA damage followed by cell cycle arrest and apoptosis in *C. albicans.* Considering the excellent anti-*Candida* potential and as it is Generally Recognized as Safe (GRAS) by the Food and Drugs Administration (FDA), toxicity concern may not arise and thus menthol may find use in antifungal chemotherapy alone or as an adjuvant.

## Figures and Tables

**Table 1:** Primers for real-time qPCR analysis of selected genes.

Gene	Forward (5–3)	Reverse (5–3)	Amplicon size
KRE9	CGGATCAAGCTTCAGGATTT	CATTTGCATTGGTGCGTATC	107
RPL11	TCCCAAAATGTTATGCGTGA	CTAAAACTTTGGCGGCTCTG	100
CDC37	GTGGTGTTATCGGTATCAGTGG	CTTGATTGGCAGTTTCATGTG	90
GAPDH	CGGTCCATCCCACAAGGA	AGTGGAAGATGGGATAATGTTACCA	—

**Table 2:** Inhibition of planktonic growth (A) of *Candida albicans* isolates and strains (*n* = 16), biofilm progression (development and maturation) of *C. albicans* (ATCC 10231) (B) by menthol alone and in combination with FLC (C).

Assay	Planktonic growth					
	Number of isolates					
*A. Broth micro dilution assay*	MIC^*∗*^ of menthol (mM)	Susceptible (*N* = 8)	S-DD (*N* = 2)	Resistant (*N* = 6)

	1.79	3	—	2

	3.58	5	2	4

B. Biofilm assay	Biofilm formation (%)

Menthol (mM)	0	3.58	7.16	14.32	28.65	57.3

Developing biofilm	100 ± 0	21 ± 9.24	14 ± 0.97	13 ± 0.65	12 ± 1.14	12 ± 0.32

Mature biofilm	100 ± 0	45 ± 11	30 ± 5.17	25 ± 2.08	25 ± 3.78	25 ± 7.02

*C. Synergistic assay*

*C1. Planktonic growth (MIC)*

Combinations	MIC FLZ (mg/L)	Menthol (mM)	FICs FLZ	Menthol	FICI	Interaction

FLZ alone	128	0.0	1.0	0.0	1.0	NI

Menthol + FLZ	8.0	0.44	0.062	0.12	0.182	S

Menthol alone	0.0	3.58	0.0	1.0	1.0	NI

*C2. Developing biofilm (MIC90)*

FLZ alone	≥256	0.0	1.0	0.0	1.0	NI

Menthol + FLZ	8.0	1.79	0.031	0.062	0.093	S

Menthol alone	0.0	28.65	0.0	1.0	1.0	NI

*C3. Matured biofilm (MIC70)*

Menthol + FLZ	8.0	1.79	0.031	0.031	0.093	S

Menthol alone	0.0	28.65	0.0	1.0	1.0	NI

^*∗*^MIC of menthol is fungicidal; thus, MIC and minimum fungicidal concentration (MFC) is same. S-DD: susceptible dose-dependent to fluconazole (According to CLSI M27 A3). ^*∗*^Stage of development: Developing biofilm = 24 h. Mature biofilm = 48 h. FIC = fractional inhibitory concentrations. FICI = ∑FICs. FLZ = fluconazole. (FICI value ≤ 0.5 = Synergy. FICI ≥0.5- ≤4.0 = No interaction and FICI ≥4 = antagonistic [22].

**Table 3:** Effect of menthol on *C. albicans* cell cycle.

Phases of cell cycle	*Cells (%)*		
	Apoptotic control	Untreated	Menthol (0.5 mM)
G1	0.1	17	5
G0-G1	3	13	8
S	2	12	15
G2-M	94	57	71

## Data Availability

All our data were included in our MS in tables and figures.

## References

[1] Kullberg B. J., Arendrup M. C. (2015). Invasive candidiasis. *New England Journal of Medicine*.

[2] Hospenthal D. R., Rinaldi M. G. (2015). *Diagnosis and Treatment of Fungal Infections*.

[3] Villa S., Hamideh M., Weinstock A. (2020). Transcriptional control of hyphal morphogenesis in *Candida albicans*. *FEMS Yeast Research*.

[4] Cdc A. (2013). Antibiotic resistance threats in the United States. *Current*.

[5] Guevara-Lora I., Bras G., Karkowska-Kuleta J. (2020). Plant-derived substances in the fight against infections caused by *Candida* species. *International Journal of Molecular Sciences*.

[6] Abualhasan M. N., Zaid A. N., Jaradat N., Mousa A. (2017). GC method validation for the analysis of menthol in suppository pharmaceutical dosage form. *International Journal of Analytical Chemistry*.

[7] Silva H. (2020). A descriptive overview of the medical uses given to mentha aromatic herbs throughout history. *Biology*.

[8] Kamatou G. P. P., Vermaak I., Viljoen A. M., Lawrence B. M. (2013). Menthol: a simple monoterpene with remarkable biological properties. *Phytochemistry*.

[9] Craighead D. H., Alexander L. M. (2016). Topical menthol increases cutaneous blood flow. *Microvascular Research*.

[10] Samarasekera R., Weerasinghe I. S., Hemalal K. P. (2008). Insecticidal activity of menthol derivatives against mosquitoes. *Pest Management Science*.

[11] Trombetta D., Castelli F., Sarpietro M. G. (2005). Mechanisms of antibacterial action of three monoterpenes. *Antimicrobial Agents and Chemotherapy*.

[12] Tanhaeian A., Sekhavati M. H., Moghaddam M. (2020). Antimicrobial activity of some plant essential oils and an antimicrobial-peptide against some clinically isolated pathogens. *Chemical and Biological Technologies in Agriculture*.

[13] Dambolena J. S., López A. G., Rubinstein H. R., Zygadlo J. A. (2010). Effects of menthol stereoisomers on the growth, sporulation and fumonisin B1 production of Fusarium verticillioides. *Food Chemistry*.

[14] Zore G. B., Thakre A. D., Rathod V., Karuppayil S. M. (2011). Evaluation of anti-*Candida* potential of geranium oil constituents against clinical isolates of *Candida albicans* differentially sensitive to fluconazole: inhibition of growth, dimorphism and sensitization. *Mycoses*.

[15] Devkatte A. N., Zore G. B., Karuppayil S. M. (2005). Potential of plant oils as inhibitors of *Candida albicans* growth. *FEMS Yeast Research*.

[16] Thakre A. D., Mulange S. V., Kodgire S. S., Zore G. B., Karuppayil S. M. (2016). Effects of cinnamaldehyde, ocimene, camphene, curcumin and farnesene on *Candida albicans*. *Advances in Microbiology*.

[17] Thakre A., Zore G., Kodgire S. (2018). Limonene inhibits *Candida albicans* growth by inducing apoptosis. *Medical Mycology*.

[18] Wayne P. (2008). *Reference Method for Broth Dilution Antifungal Susceptibility Testing of Yeasts; Approved Standard*.

[19] Zore G. B., Thakre A. D., Jadhav S., Karuppayil S. M. (2011). Terpenoids inhibit *Candida albicans* growth by affecting membrane integrity and arrest of cell cycle. *Phytomedicine*.

[20] Thakre A., Jadhav V., Kazi R. (2020). Oxidative stress induced by piperine leads to apoptosis in *Candida albicans*. *Medical Mycology*.

[21] Ramage G., Wickes B. L., López-Ribot J. L. (2007). Inhibition on *Candida albicans* biofilm formation using divalent cation chelators (EDTA). *Mycopathologia*.

[22] Odds F. C. (2003). Synergy, antagonism, and what the chequerboard puts between them. *Journal of Antimicrobial Chemotherapy*.

[23] Chen J., Jiang Q.-D., Chai Y.-P., Zhang H., Peng P., Yang X.-X. (2016). Natural terpenes as penetration enhancers for transdermal drug delivery. *Molecules*.

[24] Yang W., Chen X., Li Y., Guo S., Wang Z., Yu X. (2020). Advances in pharmacological activities of terpenoids. *Natural Product Communications*.

[25] Biswas S., Van Dijck P., Datta A. (2007). Environmental sensing and signal transduction pathways regulating morphopathogenic determinants of *Candida albicans*. *Microbiology and Molecular Biology Reviews*.

[26] Shiradhone A. B., Ingle S. S., Zore G. B. (2018). Microenvironment responsive modulations in the fatty acid content, cell surface hydrophobicity, and adhesion of *Candida albicans* cells. *Journal of Fungi*.

[27] Khare P., Chauhan A., Kumar V. (2019). Bioavailable menthol (transient receptor potential melastatin-8 agonist) induces energy expending phenotype in differentiating adipocytes. *Cells*.

[28] Lohse M. B., Gulati M., Johnson A. D., Nobile C. J. (2018). Development and regulation of single-and multi-species *Candida albicans* biofilms. *Nature Reviews Microbiology*.

[29] Cavalheiro M., Teixeira M. C. (2018). *Candida* Biofilms: threats, challenges, and promising strategies. *Frontiers of Medicine*.

[30] Rodriguez D. L., Quail M. M., Hernday A. D., Nobile C. J. (2020). Transcriptional circuits regulating developmental processes in *Candida albicans*. *Frontiers in Cellular and Infection Microbiology*.

[31] Mayer F. L., Wilson D., Hube B. (2013). *Candida albicans* pathogenicity mechanisms. *Virulence*.

[32] Xie Z., Thompson A., Sobue T. (2012). Candida albicans biofilms do not trigger reactive oxygen species and evade neutrophil killing. *Journal of Infectious Diseases*.

[33] Rosenberg A., Ene I. V., Bibi M. (2018). Antifungal tolerance is a subpopulation effect distinct from resistance and is associated with persistent candidemia. *Nature Communications*.

[34] Oz M., El Nebrisi E. G., Yang K.-H. S., Howarth F. C., Al Kury L. T. (2017). Cellular and molecular targets of menthol actions. *Frontiers in Pharmacology*.

[35] Kim S.-H., Lee S., Piccolo S. R. (2012). Menthol induces cell-cycle arrest in PC-3 cells by down-regulating G2/M genes, including polo-like kinase 1. *Biochemical and Biophysical Research Communications*.

